# Letter chunk frequency does not explain morphological masked priming: Affix frequency in masked priming

**DOI:** 10.3758/s13423-021-02010-y

**Published:** 2021-11-05

**Authors:** Mara De Rosa, Davide Crepaldi

**Affiliations:** Department of Cognitive Neuroscience, International School for Advanced Studies (SISSA), Via Bonomea 265, 34136 Trieste, Italy

**Keywords:** Morphology, Masked priming, Statistical learning, Frequency, Interpretability

## Abstract

Research on visual word identification has extensively investigated the role of morphemes, recurrent letter chunks that convey a fairly regular meaning (e.g., *lead-er-ship*). Masked priming studies highlighted morpheme identification in complex (e.g., *sing-er*) and pseudo-complex (*corn-er*) words, as well as in nonwords (e.g., *basket-y*). The present study investigated whether such sensitivity to morphemes could be rooted in the visual system sensitivity to statistics of letter (co)occurrence. To this aim, we assessed masked priming as induced by nonword primes obtained by combining a stem (e.g., *bulb*) with (i) naturally frequent, derivational suffixes (e.g., *-ment*), (ii) non-morphological, equally frequent word-endings (e.g., *-idge*), and (iii) non-morphological, infrequent word-endings (e.g., *-kle*). In two additional tasks, we collected interpretability and word-likeness measures for morphologically-structured nonwords, to assess whether priming is modulated by such factors. Results indicate that masked priming is not affected by either the frequency or the morphological status of word-endings, a pattern that was replicated in a second experiment including also lexical primes. Our findings are in line with models of early visual processing based on automatic stem/word extraction, and rule out letter chunk frequency as a main player in the early stages of visual word identification. Nonword interpretability and word-likeness do not affect this pattern.

Reading is a critical skill in our everyday life, and for skilled readers the processing of linguistic input unfolds rapidly and effortlessly. In search for the building blocks at the basis of such phenomenon, the literature is rich in studies placing special consideration on morphemes, the smallest meaning-bearing units in language (Bloomfield, 1933). Morphemes introduce a fairly regular form-to-meaning mapping, and thus provide predictable patterns that could be efficiently exploited for lexical processing (e.g., Taft & Forster, 1976; Bybee, 1988; Castles, Rastle, & Nation, 2018).

Consistent evidence encompassing many languages and experimental paradigms suggests that morphology does indeed play a role during the earliest stages of visual word identification (for reviews, see Giraudo & Voga, 2014; Rastle & Davis, 2008), even if there is little consensus on the fundamental mechanisms that are in place at such level of processing (Amenta & Crepaldi, 2012). Specifically, results from masked priming studies (Forster & Davis, 1984) indicate that embedded stems are indeed recognized within their derived forms (e.g, *sing* in *singer*). The strong and consistent facilitation elicited by such prime-target pairs is larger than the priming elicited by pairs that merely share some orthographic overlap (e.g., *twin* and *twinkle*), suggesting a morphological locus for the effect. Notably, a more controversial contrast is offered by words like *corner*, which under specific experimental conditions seem to undergo morphological analysis, leading to the somewhat counter-intuitive recognition of an embedded “stem” (i.e., *corn* within *corner*, Longtin, Segui, & Hallé, 2003; Rastle, Davis, & New, 2004).

The existence of such “morpho-orthographic” effects has been theoretically interpreted under two main types of conceptual framework. An intriguing proposal came from computational work (e.g., Baayen, Milin, DJurdjevic, Hendrix, & Marelli, 2011), which capitalizes on discriminative learning in the context of a mapping effort between orthography and semantics, and provides evidence for morphological effects without explicit morpheme representations (i.e., *-er*). Crucially, morpho-orthographic effects are here considered a by-product of residual morpheme interpretability in opaque words, which is often supported by diachronically genuine relationships (e.g., *archer* and *arch*, from the Latin *arcus*) that could carry some degree of semantic transparency. Nevertheless, such account has been experimentally challenged by data exhibiting morpho-orthographic effects in unambiguously semantically opaque prime-target pairs (Beyersmann et al., 2016).

Alternatively, several theoretical accounts postulate morpho- orthographic processing to depend on explicit levels of representation, with earlier proposals offering morphology itself as a primary organizational principle (e.g., Rastle & Davis, 2003; Taft, 1994). However, most of these models do not reckon with recent evidence coming from nonword primes, which produce strong facilitation even without a complete morphological structure (e.g., Beyersmann, Casalis, Ziegler, & Grainger, 2015; Beyersmann, Cavalli, Casalis, & Colé, 2016; Hasenäcker, Beyersmann, & Schroeder, 2016; Grainger & Beyersmann, 2020).

More recently, the emphasis switched away from morphology *per se* and more attention was put on letter statistics (e.g., Crepaldi, Rastle, & Davis, 2010; Grainger & Ziegler, 2011). The key insight in this respect is that morphemes also constitute recurrent letter strings in the written language: because they carry some meaning, morphological stems and chunks like *-ment*, *-ness* or *-er* occur relatively frequently across several different words, and therefore might become fairly salient units also from a merely orthographic, statistical point of view.

In support of this conjecture, sensitivity to statistical regularities is known to be an extremely powerful cognitive resource recruited in information processing in general (for recent reviews, see Frost, Armstrong, & Christiansen, 2019; Armstrong, Frost, & Christiansen, 2017; Aslin, 2017; Christiansen, 2019; Thiessen, Kronstein, & Hufnagle, 2013), and in reading in particular (e.g., Arciuli & Simpson, 2012; Treiman, Gordon, Boada, Peterson, & Pennington, 2014; Sawi & Rueckl, 2019; Schubert, Cohen, & Fischer-Baum, 2020; Chetail, 2015). Recent experimental evidence in the context of the statistical learning framework substantiated the relationship between sublexical units and statistical patterns (e.g., Chetail, 2017; Lelonkiewicz, Ktori, & Crepaldi, 2020). In particular, Lelonkiewicz et al., (2020) modelled morpheme learning in an artificial lexicon where the only possible cues were characters’ statistics. When passively exposed to strings of pseudoletters, skilled readers rapidly developed sensitivity to patterns of affixation on the basis of their frequency of occurrence. Crucially, the carved prefix and suffix-like units could not benefit from phonological or semantic information, and yet they exhibited some typical aspects of affix processing (i.e., positional constraints, Crepaldi et al., 2010). Frequency of occurrence is indeed a pivotal feature for language learning (e.g., Ellis, 2002), and frequency effects have often been considered proxies of learned representations in lexical decision studies (Baayen, Wurm, & Aycock, 2007; Burani & Thornton, 2003; Colé, Beauvillain, & Segui, 1989; Taft 1979, 2004). These considerations strengthen the idea that the “corner-corn” effect – and, more generally, morpho-orthographic chunking – might be based on the letter statistics that morphemes typically show, and particularly their frequency of occurrence. Some investigation of this hypothesis was carried out by Beyersmann, Ziegler, and Grainger (2015), who failed to observe a cluster frequency effect in a letter search task. This clearly speaks against the idea that the frequency of letter chunks is a primary driver during visual word identification. However, letter search is not the paradigm where morpho-orthographic effects are typically found, and the efficacy of this task in addressing morphological processing has been recently questioned (Hasenäcker, Ktori, & Crepaldi, 2021).

To address these issues, we carried out a masked priming, lexical decision experiment with nonword primes. Specifically, we assessed the facilitation elicited by morphologically structured primes (e.g.,*bulb-er*), and by non-morphologically structured nonwords whose endings were either as frequent (e.g., *bulb-le*) or consistently less frequent (e.g., *bulb-ew*) than suffixes. This manipulation allowed us to determine whether morpho-orthographic analysis stems from written frequency, or is rather subordinate to word-endings’ morphological status.

Morphologically structured nonwords may lend themselves to some plausible semantic interpretation (e.g., *idealike*, *heroable*; Günther & Marelli, 2020), which could in turn influence masked priming (e.g., Heathcote, Nation, Castles, & Beyersmann, 2018). To test for such possibility, after the main task participants were asked to provide interpretability ratings for the morphologically structured nonword primes. If facilitation could be explained by such interpretability ratings, this would suggest a role for the genuine morphological status of the suffixes/word-endings, rather than for their frequency.

Additionally, on the grounds of their structure, morphological nonwords can trigger activation into the lexical-semantic system, as indexed by the difficulty with which they are rejected in lexical decision tasks (*morpheme interference effect*; Taft & Forster, 1975; Yablonski & Ben-Shachar, 2016; Crepaldi et al., 2010, Beyersmann, Mousikou, Javourey-Drevet, Schroeder, Ziegler, & Grainger, 2020) and by their speed of processing in reading aloud (Burani, Arduino, & Marcolini, 2006). Therefore, an additional task was devised to estimate the interference induced by each morphological nonword, which could also affect masked priming; such eventual correlation might shed some light on how the masked priming results relate to the lexical-semantic system in its entirety.

## Experiment I

### Methods

#### Participants

Fifty-six Italian native speakers (19 males; age: *M* = 24.86, *SD* = 3.59) took part in the study after giving written informed consent. All participants had normal or corrected-to-normal vision and no history of linguistic or neurological impairment. They were compensated for their time, effort and travel expenses with a monetary reimbursement. The study was approved by the Ethics Committee at the International School for Advanced Studies, where participants were tested.

#### Masked priming lexical decision task

##### Materials

Twenty-nine derivational Italian suffixes (e.g., *-mento*, English: *-ment*) were selected to have a fairly high token frequency (*M* = 4.84, *SD* = 0.61; length, *M* = 3.82, *SD* = 0.86). Meaningless word-endings (e.g., *-erso*) of comparable length (*M* = 3.87, *SD* = 0.33) were selected to construct a group of highly frequent (HF, *M* = 4.94, *SD* = 0.67) and one of infrequent (LF, *M* = 2.33, *SD* = 0.31) letter clusters, resulting in three sets of existing word-endings. Crucially, suffixes and highly frequent word-endings were of comparable token frequency, and differed solely on the basis of their morphological status; conversely, the low-frequency set of letter chunks consistently differed in terms of written frequency from the other two groups.

Seventy-eight nouns (e.g., *radio*, length: *M* = 5.81; *SD* = 1.12) were then selected as target words. The set of items was constructed to be fairly homogeneous in terms of written frequency (*M* = 2.82, *SD* = 0.45) and orthographic neighborhood size (OLD20, Yarkoni, Balota, & Yap, 2008; *M* = 1.36, *SD* = 0.31). The stem of each target word was then combined with one letter cluster from each group of word-endings, thus resulting in 78 primes in each of three conditions: morphologically complex (Morph, length: *M* = 8.70, *SD* = 1.28; OLD20: *M* = 2.60, *SD* = 0.49; e.g., *radieria*), orthographic - high frequency (HF, length: *M* = 8.75, *SD* = 1.13; OLD20: *M* = 2.90, *SD* = 0.49; e.g., *radierso*) and orthographic - low frequency (LF, length: *M* = 8.87, *SD* = 1.08; OLD20: *M* = 3.14, *SD* = 0.44; e.g., *radieffa*). For each prime in each condition, an unrelated prime was obtained by combining the same ending to an unrelated stem (e.g., *bomberia*-*radio* acted as a control for *radieria-radio*). The unrelated primes were matched for both length and orthographic neighborhood size (Morph, length: *M* = 8.70, *SD* = 1.27; OLD20: *M* = 2.61, *SD* = 0.51; e.g., *bomberia*), orthographic - high frequency (HF, length: *M* = 8.73, *SD* = 1.13; OLD20: *M* = 2.88, *SD* = 0.51; e.g., *bomberso*); orthographic - low frequency (LF, length: *M* = 8.87, *SD* = 1.08; OLD20: *M* = 3.14, *SD* = 0.44; e.g., *bombeffa*). Seventy-eight nonword targets were then obtained by substituting one consonant from an Italian existing simple word and were adopted as filler items (e.g., *tafolo* from the word *tavolo*, table). Each nonword target was matched with a prime, constructed by adding the three-conditions clusters to a related or unrelated nonword base (e.g., *tafolmento*-*tafolo*, *taflement*-*tafle*, or *ritelmento*-*tafolo*, *retelment*-*tafle*).

The overall stimulus set was split into six sublists, following a within-item, within-subject Latin Square design, hence guaranteeing that participants were exposed to all conditions without seeing any target more than once.

##### Procedure

Each trial began with a 500-ms string of hash marks, followed by the lowercase prime (50 ms) and the uppercase target, which remained on screen for 1500 ms or until response. Consecutive trials were separated by an interval jittered around 1500 ms. Sessions also comprised two examples, 12 practice and four warm-up trials, which were all excluded from the analyses.

#### Subsidiary tasks

##### Materials

Morphological nonword primes, both related and unrelated, were further investigated through (i) an overt rating and (ii) an unprimed lexical decision task. These tasks were conceived to estimate any specific processing induced by the morphological nonword primes, either at an explicit level (i.e., through overt rating) or implicitly, via the delay on correct rejections in a lexical decision (*morpheme interference effect*; Taft & Forster, 1975; Yablonski & Ben-Shachar, 2016; Crepaldi et al., 2010; Beyersmann et al., 2020).

The rating task included for each participant the set of 26 nonwords that served as primes in the main task for that given participant; no filler items were used. Participants were asked to judge how easily they would be able to attribute a meaning to each nonword on a 1 (not interpretable at all) to 7 (easily interpretable) scale.

For the lexical decision task, each participant was shown the same 26 nonwords that served as primes in the main task. We additionally selected 26 derived Italian words to match the features and the structure of the morphological nonwords (e.g., *amicizia*, *friendship*; length: *M* = 8.72, *SD* = 1.27; OLD20: *M* = 2.32, *SD* = 0.54; written frequency: *M* = 1.83, *SD* = 0.87). The stimulus set comprised also 13 morphologically simple Italian words (e.g., *anguria*, *watermelon*; length: *M* = 8.77, *SD* = 1.83; OLD20: *M* = 2.00, *SD* = 0.69; written frequency: *M* = 2.64, *SD* = 0.61), and 13 nonwords obtained by substituting one consonant from an Italian existing simple word (e.g., *pafola*, from *parola*, *word*; length: *M* = 8.46, *SD* = 1.56; OLD20: *M* = 2.55, *SD* = 0.55).

##### Procedure

The lexical decision trials began with a central fixation cross presented for 500 ms, followed by the uppercase target stimulus presented for 2000 ms or until response; consecutive trials were separated by a jittered interval of 1500 ms. In the rating task, the morphologically structured nonwords were presented one at a time in lowercase, and remained on the screen until response, without time constraints.

#### Apparatus

Participants were run individually in a soundproof experimental booth, seated at approximately 80 cm in front of a BenQ XL2720Z monitor (27”, 1920x1080 pixels, 144 Hz). They provided YES responses with their dominant hand (7 left-handed) through a Cedrus Response Pad RB-730. The experiment was administered via PsychToolbox-3 ((Brainard, 1997); http://psychtoolbox.org/) on MATLAB R2015b (The Mathworks) in a Windows environment; stimuli were rendered as strings of Arial characters (32 pt), presented in white over a black background at the center of the screen.

#### Data analysis

##### Main task (masked priming)

One target with an overall error rate over 30% was excluded from the analysis (*prua*, *prow*). One subject was below 80% in accuracy on nonword trials and was thus discarded. Anticipatory responses of less than 200 ms (1.66%), no-response trials (1.65%) and incorrect responses (3.64%) were also removed. Linear mixed-effects models with crossed random intercepts for subjects and target words (Baayen, Davidson, & Bates, 2008), as implemented in *lme4* (Bates, Sarkar, Bates, & Matrix, 2007) and *lmerTest* (Kuznetsova, Brockhoff, & Christensen, 2017), were employed as the primary statistical tool.

The main analysis for the masked priming task was performed on inverse-transformed reaction times. The effects of interest were Relatedness (i.e., related vs. unrelated primes) and Condition (i.e., affixes, high-frequency endings and low-frequency endings), as well as their mutual interaction. In addition, we tested whether Relatedness interacted with the item-level metrics for interpretability and morpheme interference (see below); as non-morphemic primes do not have these metrics at all, this further analysis was carried out only on morphological primes. Other fixed effects were included if they significantly improved the model’s goodness of fit in a backward step-wise model selection procedure, resulting in the inclusion of log-transformed target frequency, as well as accuracy and reaction time on the preceding trial.

Non-significant effects were further explored through Bayes Factor (BF) analyses (Dienes, 2014) as implemented in the R package BayesFactor (Morey, Rouder, & Jamil, 2015). In particular, the preferred model (that is, the one expressing the hypothesis under study) was compared against models without the effects of interest, thus providing quantifiable evidence in support of H1 or H0.

##### Subsidiary tasks (ratings and morpheme interference)

Rating scores for the morphologically complex nonwords were transformed into within-subject z-scores:

$$ z_{ij} = \frac{r_{ij} - M(r_{j})}{SD(r_{j})} $$

where *r*_*ij*_ is the rating given to item *i* by subject *j*, and *M*(*r*_*j*_) and *SD*(*r*_*j*_) are the mean and standard deviation across all ratings offered by subject *j*. These z-scores were then averaged across participants for each item, thus generating an item-specific metric for interpretability.

Reaction times for correct nonword rejections in the lexical decision task were again analyzed through linear mixed models, with Condition, target length and OLD20 as fixed effects, and crossed random intercepts for items and participants. Before modelling, we removed data from one participant with accuracy below 60%, no-response trials (0.36%) and incorrect responses (11.19% of total). Because all the other main sources of variance across nonwords were taken up by the fixed effects (e.g., length, orthographic neighbourhood size), the random intercept for items was taken as an index of how much more (or less) morpheme interference any given nonword brings about.

These two indices were used to model reaction times in the masked priming task for morphologically complex primes (see above).

## Results

**Fig. 1:**
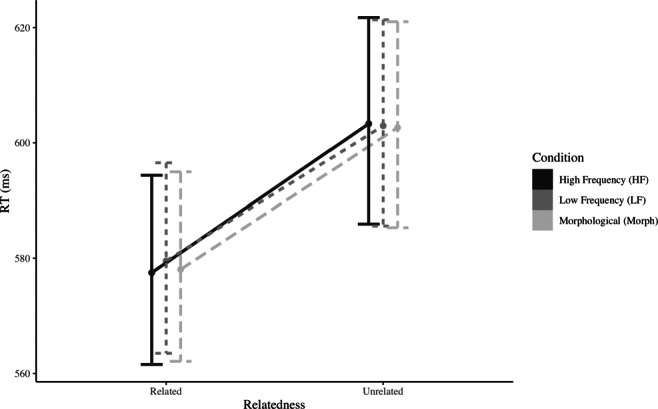
Model estimates of reaction times in the masked priming lexical decision task. Error bars depict 95% confidence intervals

The pattern of results in the main priming task is illustrated in Fig. 1. The analysis of response times in the masked priming task resulted in a main effect of Relatedness (*χ*^2^ = 46.926,*p* < 0.001), indicating that related primes induced faster responses compared to their unrelated baseline. However, we observe no effect of Condition (*χ*^2^ = 0.313,*p* = 0.855) nor an interaction between Condition and Relatedness (*χ*^2^ = 0.547,*p* = 0.761). A Bayes Factor analysis provided strong evidence against a model including an interaction (0.006 ± 2.28*%*) or a main effect of Condition (0.002 ± 2.5*%*). Therefore, the strong priming hereby observed is not influenced by either the frequency or the morphological status of the word-endings. Instead, the mere presence of the target stem in the prime seems enough to result in a consistent facilitation.

**Fig. 2:**
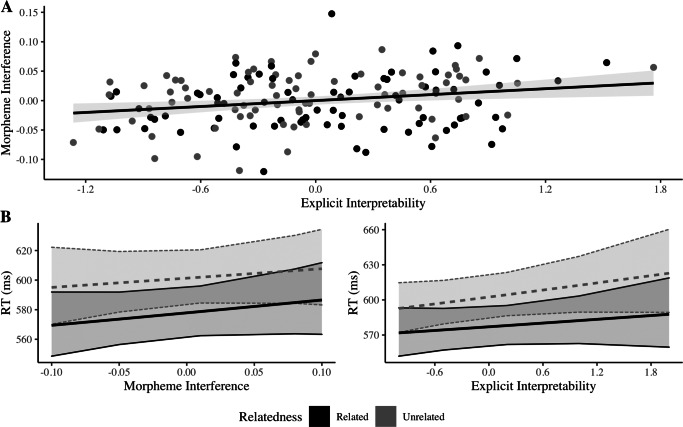
(A) Correlation between Morpheme Interference and Explicit Interpretability indices; (B) Estimated effect of both indices on masked priming reaction times. RTs increase slightly with growing Morpheme Interference/Interpretability (although the effect doesn’t reach significance), but priming remains clearly constant. Shaded area depict 95% confidence intervals

The morpheme interference task revealed a strong effect of Condition (*χ*^2^ = 12.576,*p* = 0.0004), with morphological nonwords eliciting longer reaction times for correct rejections. The index obtained from this task yielded a rather moderate, albeit significant, correlation with the explicit ratings (*r* = 0.23,*t*(152) = 2.881,*p* = 0.004, Fig. 2A), thus indicating that both indices pinpoint distinct, although somewhat related phenomena. However, neither index affected priming (Fig. 2B). The analysis of reaction times induced by morphologically complex primes showed a non-significant main effect of Morpheme Interference (*χ*^2^ = 0.449, *p* = 0.502), and a lack of interaction with Relatedness (*χ*^2^ = 0.059, *p* = 0.806). A similar pattern was yielded by the model considering Explicit Interpretability (*χ*^2^ = 2.088,*p* = 0.148, interaction with Relatedness: *χ*^2^ = 0.215,*p* = 0.642). Once again, these null results were corroborated through Bayes Factor analyses (Morpheme Interference: 0.042 ± 2.09*%*; Explicit Interpretability: 0.022 ± 1.71*%*).

## Ad interim discussion

The present experiment aimed at disentangling the role of co-occurrence regularities in morpho-orthographic effects. We combined existing Italian stems with genuine suffixes, high-frequency and low-frequency word-endings, and examined the facilitation elicited by each type of nonword in a masked priming, lexical decision study. A strong priming effect emerged independently of either frequency or the morphological nature of word-endings. Coherently, the facilitation elicited by morphological nonwords (e.g., *heroable*) did not depend on rating-based semantic interpretability nor on how much morpheme interference effect they caused in a plain lexical decision task.

Following the suggestion of an anonymous reviewer, we tried to replicate these results in a second masked priming experiment that also included *corner-corn* prime-target pairs. The aim was twofold: to offer a self-replication, and to assess whether the inclusion of lexical primes could affect the observed pattern of facilitation.

## Experiment II

### Methods

#### Participants

Forty-five Italian native speakers (14 males; age: *M*= 26.08, *SD*= 4.18) took part in the study after giving written informed consent. All participants had normal or corrected-to-normal vision and no history of linguistic or neurological impairment.

#### Materials

In addition to the three conditions explored in Experiment 1, we included prime-target pairs of pseudo-derived words (e.g., *corner*) priming their pseudo-stem (e.g., *corn*) – the classic morpho-orthographic priming condition (Rastle et al., 2004; Longtin et al., 2003). The inclusion of word primes did not allow to construct a stimulus set that would satisfy all the relevant formal constraints in a within-item design; consequently, we made use of different target words in the four conditions. More specifically, 40 words (e.g., *rosa*, English: *rose*) were selected for each condition, matched for length, frequency and orthographic neighbourhood size (Table 1). The sets of targets were different but comparable across experiments.

**Table 1:** Target features (mean and standard deviation) in Experiment 1

Condition	Frequency	Length	OLD20
High Frequency (HF)	3.645 (0.738)	5.05 (1.011)	1.141 (0.282)
Low Frequency (LF)	3.615 (0.722)	5.025 (1)	1.176 (0.296)
Morphological (Morph)	3.611 (0.786)	5.025 (0.862)	1.176 (0.316)
Opaque - Word (Op)	3.614 (0.765)	4.975 (0.768)	1.124 (0.228)

Each target word was then paired with both a related and an unrelated prime, coherently with the assigned condition. Targets in the morpho-orthographic condition were paired with pseudo-derived word primes (e.g., *cervello-cervo*, comparable to *corner-corn* in English). Nonword primes were obtained with the same logic of Experiment 1, that is, by combining the stems of the selected targets with suffixes (e.g., *-eria*; frequency: *M* = 5.10, *SD* = 0.47; length: *M* = 3.9, *SD* = 0.71), highly frequent, meaningless word-endings (e.g., *-upe*; frequency: *M* = 5.08, *SD* = 0.32; length: *M* = 3.82, *SD* = 0.39) and low frequency, meaningless word-endings (e.g.,*-iaba*; frequency: *M* = 2.60, *SD* = 0.82; length: *M* = 3.85, *SD* = 0.36). Overall, we constructed one set of word primes and three sets of nonword primes, which were matched for length and orthographic neighbourhood size and paired with carefully constructed unrelated baselines (Table 2).

**Table 2:** Prime features (mean and standard deviation) in Experiment 1

Condition	Relatedness	Frequency	Length	OLD20
High Frequency (HF)	Related	0	7.85 (1.231)	2.466 (0.552)
High Frequency (HF)	Unrelated	0	7.875 (1.223)	2.461 (0.522)
Low Frequency (LF)	Related	0	7.825 (1.196)	2.752 (0.495)
Low Frequency (LF)	Unrelated	0	7.875 (1.223)	2.692 (0.582)
Morphological (Morph)	Related	0	7.85 (1.231)	2.244 (0.574)
Morphological (Morph)	Unrelated	0	7.85 (1.231)	2.215 (0.637)
Opaque - Word (Op)	Related	2.624 (0.767)	7.85 (1.231)	1.742 (0.442)
Opaque - Word (Op)	Unrelated	2.657 (0.902)	7.825 (1.238)	1.709 (0.432)

As a result, the stimulus set was made up of 160 target words, each paired with a related and an unrelated prime, which were rotated across participants in a between-item, within-subject Latin Square design; note that this approach implies only two rotations, compared to the six rotations adopted in Experiment 1.

One hundred and sixty nonwords were then obtained by substituting a consonant from an existing Italian simple word (e.g., *fuolo* from the word *fuoco*, *fire*). These items were used as NO-response targets in filler trials. Following the approach adopted in Experiment 1, each nonword target was matched with a nonword prime, constructed by adding the same clusters as in experimental conditions to a related or unrelated nonword base (e.g., *fuolerpe*).

#### Procedure

The trial timeline was identical to Experiment 1, with the sole exception of primes being presented more briefly (42 ms) to further ensure their effective masking.

#### Data analysis

The analysis approach was identical to Experiment 1 in all details.

One subject was below 80% in accuracy on nonword trials and was thus discarded. No-response trials (0.32%) and incorrect responses (2.51%) were also removed. The final model included log-transformed frequency, orthographic neighbourhood size and length of the targets, prime length as well as trial number within the session, accuracy and reaction time on the preceding trial.

## Results

**Fig. 3:**
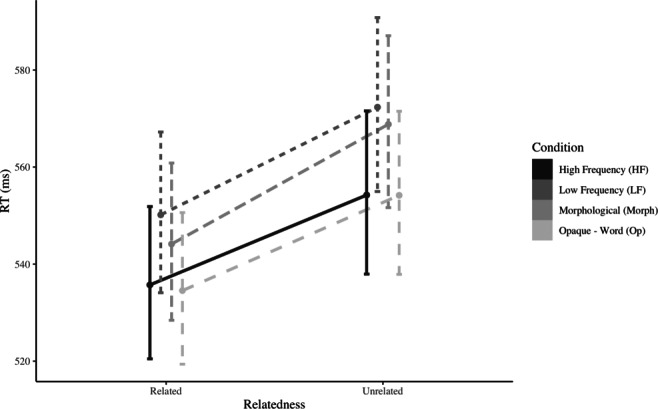
Model estimates of reaction times in Experiment 1. Error bars depict 95% confidence intervals

The results obtained from Experiment 1 are displayed in Fig. 3, with primes from all conditions showing a facilitatory pattern (main effect of Relatedness, (*χ*^2^ = 42.372, *p* < 0.001). Critically, we replicate the lack of an interaction between Condition and Relatedness (*χ*^2^ = 1.151, *p* = 0.765). Such result is corroborated by a Bayes Factor analysis, which provides strong evidence against the model including an interaction factor (0.00082 ± 3.09*%*).

## General discussion

The present study aimed at establishing the relationship between morpho-orthographic effects and letter co-occurrence regularities. Since morphemes are also frequent letter clusters, we combined existing Italian stems with genuine suffixes, high-frequency and low-frequency word-endings, and examined the facilitation elicited by each type of nonword in a masked priming, lexical decision study. A strong priming effect emerged independently of either frequency or morphological nature of word-endings. Such pattern of results was further corroborated through an additional experiment including lexical primes with semantically opaque relations to their targets (e.g., *corner-corn*). Coherently, the facilitation elicited by morphological nonwords (e.g., *heroable*) did not depend on rating-based semantic interpretability nor on how much morpheme interference effect they caused in a plain lexical decision task.

The main results of the masked priming task are in line with a wealth of studies on nonword primes, which consistently reported solid facilitation effects independently of the presence of an affix (that is, with primes like *farmness*, but also with primes like *farmald*; e.g., Beyersmann et al., 2015; Beyersmann et al., 2016; Hasenäcker et al., 2016; Hasenäcker, Beyersmann, & Schroeder, 2020; Morris, Porter, Grainger, & Holcomb, 2011; McCormick, Brysbaert, & Rastle, 2009; Beyersmann & Grainger, 2018; Heathcote et al., 2018). It is noteworthy that such a pattern is different from the solidly replicated evidence coming from word primes, in which the presence of both a (pseudo-) stem and a (pseudo-)affix is necessary to elicit facilitation (*corner* primes *corn*, but *brothel* does not prime *broth*; e.g., Rastle et al., 2004). This asymmetry between word and nonword primes could be quite easily accounted for by lexical competition. While nonword primes have no established representations, words might compete for activation in the lexical-semantic system: *corner* and *cornea* would be competing with *corn*, but neither *cornity* nor *cornew* would. Therefore, if morphology becomes crucial to overcome competition and win the race for activation in word priming (i.e., allowing *corn* to be activated in *corner*, but not in *cornea*), the embedded target word can be easily activated in a nonword prime by virtue of its mere presence (e.g., Grainger & Beyersmann, 2017). Such early activation of shared elements between primes and targets could arise from different mechanisms, as implemented by several models in the literature. For instance, Grainger and Beyersmann (2017) suggest that such a mechanism is deputed to the extraction of edge-aligned embedded words; on the other hand, Crepaldi et al., (2010) and Taft (2004) stipulate the existence of a pre-lexical level of representation that would capture both stems and affixes.

While the present results are largely compatible with either account of stem extraction, they clearly speak against morphology *per se* as the main driver of the early stages of visual word identification, as postulated by earlier models of pre-lexical processing (e.g., Crepaldi et al., 2010; Taft, 2004; Taft and Nguyen-Hoan, 2010). Instead, our findings are better aligned with more recent theories that emphasise orthographic, rather than morphological factors. The hypothesis at the core of the present study is that morpho-orthographic effects are mostly based on letter statistics; morphemes, among other letter chunks, are frequent letter clusters, and this is why they would be captured at this level of processing. This account is coherent with recent findings reported by Grainger and Beyersmann (2020), suggesting that in the absence of a pseudo-morphological structure, nonword priming might be affected by conditional probabilities between the identified stem and an eventual (derivational) affix.

However, the statistical regularity manipulated in the present study (i.e., word-ending frequency) did not affect morpho-orthographic priming, a result that is also consistent with previous evidence coming from letter search studies (Beyersmann et al., 2015). Despite its essential role in linguistic processing (e.g., Ellis, 2002), letter-cluster frequency should be regarded as only one possible metric of letter co-occurrence regularities. It is hence possible for other, more sophisticated metrics (such as the aforementioned conditional probability) to play a more prominent role in early visual word processing. This would in turn suggest the involvement of a different mechanism, perhaps of a predictive nature (e.g., Avarguès-Weber, Finke, Nagy, Szabó, d’Amaro, Dyer, & Fiser, 2020), as suggested by several experiments across different cognitive (see Frost et al., 2019, for a recent review on the history and interplay of co-occurrence metrics).

The primary role of statistical cues, rather than morphological status *per se*, is also widely supported by some evidence obtained in artificial lexicon studies (e.g., Lelonkiewicz et al., 2020; Chetail, 2017), in which readers were unequivocally shown to be sensitive to the frequency with which chunks of pseudo-characters co-occur. One key difference is in the nature of the tasks adopted, as well as in the experimental demands of these studies. Specifically, learning studies with artificial characters focus on unfamiliar material that becomes somewhat familiar by the end of the experiment. Since the pioneering work of Saffran, Aslin, and Newport (1996), the extraction of statistical information has in fact proven to be the bedrock behind the learning of linguistic (or pseudo-linguistic) material, with frequency of occurrence having a pivotal role during the first phases of exposure to novel stimuli. The goal of the present study was instead to uncover the role of co-occurrences within the processing of well-established units (existing letters and morphemes). It is possible, then, that while growing in familiarity and proficiency with a writing system, letter statistics contribute to create and consolidate higher-level representations, a stage that would be captured by artificial lexicon studies. Once such higher-level representations are well established, they would acquire an autonomous, rooted status, thus making the system less reliant on mere frequency (and perhaps other statistical cues, more generally); this would be the stage captured here, and in other studies with real linguistic material. The frequency of letter chunks would be critical during learning, but perhaps less so in the mature system, where those chunks have probably acquired representations on their own.

This interpretation highlights the idea that the early stages of visual word processing, even if they are indeed heavily based on language-agnostic, statistical processes, are also in constant interaction with more central, lexical representations (whenever these representations are available). This resolves the apparent conflict with data showing a role for morphological family size (Beyersmann & Grainger, 2018) and position of the embedded stem (Beyersmann, Kezilas, Coltheart, Castles, Ziegler, Taft, & Grainger, 2018) in complex nonword priming effects.

An additional result is that nonword priming is not modulated by either lexical-semantic drive (as indexed by the amount of morpheme interference), or interpretability (as gauged by overt ratings). Importantly however, metalinguistic ratings constitute only one possible operationalization of nonword interpretability. Compelling evidence for more sophisticated, model-based metrics comes from work on novel compounds (Günther & Marelli, 2020), in which semantic effects are captured through compositional processes, an approach that paves the way for a more dynamic view of meaning-combination mechanisms in novel derived forms (Amenta, Günther, & Marelli, 2020). Nevertheless, in keeping with several previous studies (e.g., Longtin & Meunier, 2005; Giraudo & Voga, 2016; Tseng, Lindsay, & Davis, 2020), the present results suggest that masked priming is not much influenced by semantic effects, at least with derived nonword primes (see, e.g., Feldman & Basnight-Brown, 2008; Feldman, Kostić, Gvozdenović, O’Connor, & del Prado Martýn, 2012, for a somewhat different perspective with word primes). More generally, these data suggest a weak relationship between the mechanisms captured by masked priming, and the lexical-semantic dynamics induced by the same nonwords when presented overtly, as target items. Such result might indicate that while masked priming taps into early processing, overt-ratings and morpheme interference are based on subsequent stages, thus calling for an integrated view of morpho-orthographic phenomena within the lexical-semantic system at large.
